# Case report: Maternal engraftment of T-cells in a patient with Severe Combined Immunodeficiency due to *DCLRE1C* pathogenic variant - diagnostic and treatment challenges

**DOI:** 10.3389/fimmu.2026.1892927

**Published:** 2026-09-30

**Authors:** Evija Peiseniece, Marija Rozevska, Viktorija Arnite, Jelena Rascon, Ginte Grubliauskaite, Inga Nartisa, Livija Bardina, Gita Taurina, Dmitrijs Rots, Natalja Kurjane

**Affiliations:** 1 Faculty of Medicine, Riga Stradiņš University, Riga, Latvia; 2 Riga Stradiņš University, Institute of Oncology and Molecular Genetics, Riga, Latvia; 3 Children’s Clinical University Hospital, Riga, Latvia; 4 Center for Pediatric Oncology and Hematology, Vilnius University Hospital Santaros Klinikos, Vilnius, Lithuania; 5 Faculty of Medicine, Vilnius University, Vilnius, Lithuania; 6 Department of Clinical Genetics, Erasmus Medical Center, Rotterdam, Netherlands; 7 Pauls Stradiņš Clinical University Hospital, Center for Clinical Immunology and Allergology, Riga, Latvia

**Keywords:** ARTEMIS deficiency, hematopoietic stem cell transplantation (HCST), IEI, maternal cell engraftment, severe combined immunodeficiency (SCID)

## Abstract

**Background:**

Severe combined immunodeficiency (SCID) is a rare but serious congenital condition caused by genetic defects that severely impair the development and function of T cells, B cells, and natural killer (NK) cells. Without early diagnosis and immune system reconstitution, SCID is usually fatal in infancy. The condition requires prompt recognition and hematopoietic stem cell transplantation (HSCT) to restore immune function. This report describes the first genetically confirmed case of SCID in Latvia – caused by a *DCLRE1C* gene pathogenic variant and diagnosed prior to the implementation of national newborn screening for SCID – complicated by disseminated tuberculosis following Bacillus Calmette-Guérin (BCG) vaccination.

**Case presentation:**

The male infant was born after an uneventful pregnancy to healthy parents with no known genetic disorders. The patient experienced recurrent bacterial respiratory infections and received the first scheduled vaccination, the BCG vaccine, at 2 months of age, later than recommended in the national immunization schedule because of parental hesitancy related to concerns about vaccine safety. Shortly after receiving the BCG vaccine, the infant developed erythroderma and a maculopapular rash, followed by pneumonia, acute bronchiolitis, and a bacterial skin infection at three months of age. Immunological evaluation revealed an atypical pattern for SCID, with CD3+ T cells at 53% (abs. 764/μL) and CD4+ T cells at 52% (abs. 748/μL), but a profound deficiency of CD8+ T cells (0.79%, abs. 11/μL). In our case, genetic analysis identified a homozygous pathogenic variant in the *DCLRE1C* gene. The presence of an XX cell line suggested maternal T-cell engraftment. The patient underwent HSCT; however, post-transplant disseminated tuberculosis required antituberculosis therapy and significantly affected the outcome.

**Conclusions:**

This case underscores the importance of early diagnosis and, specifically, early antimycobacterial prophylaxis in SCID: although the diagnosis was genetically confirmed at 4 months of age, prophylactic antimycobacterial treatment was not started until disseminated BCG disease was identified 3.5 months later, at 7.5 months of age, and this delay was followed by prolonged multidrug antimycobacterial therapy and a complicated post-transplant course. This highlights the risks of administering live vaccines before immune status is confirmed and reinforces the critical role of newborn screening programs for SCID, which Latvia began implementing on April 1, 2023.

## 1 Introduction

Severe combined immunodeficiency (SCID) comprises a heterogeneous group of diseases characterized by severe immunodeficiency, which includes disorders of T and B cell development, as well as, occasionally, NK cell development (1). SCID is among the most significant immunodeficiencies in the spectrum of inborn errors of immunity (IEI), affecting adaptive and, occasionally, innate immunity (2, 3).

IEI are categorized by the immune system compartment affected (4). A “severe” deficiency resulting from a serious immune cell defect can lead to high mortality if not diagnosed early and treated properly. Genetic testing plays a crucial role in diagnosing SCID, as inherited gene variants significantly affect the development and function of the immune system. Over 20 genes have been identified as linked to SCID (5). Phenotypically, SCID can be broadly categorized into T-B-SCID and T-B+SCID phenotypes based on B cell status, and further subdivided according to natural killer cell status (6). Individuals with SCID may exhibit a lack of lymphoid tissue, profound lymphopenia, significantly reduced or absent T- and B-cell function, and hypogammaglobulinemia (7, 8).

SCID is the only life-threatening inborn error of human immunity for which early detection is crucial, and urgent hematopoietic stem cell transplantation (HSCT) is the only treatment (9) (EBMT/ESID Inborn Errors Working Party Guidelines for Hematopoietic Stem Cell Transplantation for Inborn Errors of Immunity). Studies show that infants who undergo HSCT before 3.5 months of age have a very high survival rate of up to 94%. The survival rate is 90% for older infants with no prior infections and 82% for those with a resolved infection before HSCT (10, 11).

Patients diagnosed with SCID are extremely vulnerable to infections from early infancy and therefore require strict protective measures to minimize exposure to pathogens. Live-attenuated vaccines are contraindicated in SCID patients because of the high risk of severe, disseminated disease. In Latvia, according to the Centers for Disease Control and Prevention’s childhood vaccination calendar and the national immunization schedule, one of the first vaccines administered is the live attenuated Bacillus Calmette–Guérin (BCG) vaccine against tuberculosis. At the same time, the incidence of tuberculosis in Latvia remains higher than the average in European Union countries, resulting in a persistent risk of infection among infants and young children (12).

This epidemiological situation increases the clinical impact of routine neonatal BCG vaccination because infants with unrecognized SCID are at particularly high risk of developing severe, disseminated tuberculosis and other life-threatening infections following administration of live attenuated vaccines (13).

SCID patients experience recurrent respiratory, gastrointestinal, opportunistic, and viral infections, and persistent or severe disease can result from live-attenuated vaccines. Early detection of SCID through newborn screening using the T cell receptor excision circle (TREC) test enables prompt diagnosis. In Latvia, there is concern because legislation requires a newborn screening sample to be collected 48–72 hours after birth, with results available by the tenth day of life. Meanwhile, vaccination against tuberculosis can be administered as early as 12 hours after birth, ideally as soon as possible, to prevent exposure to potential pathogens. This means that many infants with undetected IEI may receive the vaccine before their screening results are known.

This publication presents the first reported case of SCID with maternal T-cell engraftment in Latvia and highlights the challenges of timely diagnosis and subsequent management.

## 1 Methods

The demographic and clinical data described in a case report were collected at the Children’s Clinical University Hospital, with ethical approval from the Scientific Ethics Committee of Riga Stradins University, received on January 30, 2024, No. 2-PĒK-4/142/2024, and approval from the Children’s Clinical University Hospital, received on February 27, 2024. Parents had previously signed a consent form, adhering to the principles outlined in the Declaration of Helsinki.

## 2 Case description

We report the case of a male infant with Artemis severe combined immunodeficiency (SCID) complicated by maternal T-cell engraftment and disseminated Bacillus Calmette–Guérin (BCG) disease following delayed BCG vaccination.

The first child in the family was born after an uncomplicated pregnancy to healthy, non-consanguineous Latvian parents with no known family history of primary immunodeficiency or other inherited disorders. Delivery was by emergency cesarean section at 41 weeks of gestation due to prolonged labor. The neonatal course was uneventful, and he was discharged from the maternity hospital on the third day of life.

The previously healthy infant received the BCG vaccine at 2 months of age, later than the national recommendation, due to parental hesitancy over vaccine safety, which delayed immunization.

No immediate local or systemic adverse reactions were observed. Over the following weeks, he developed recurrent bacterial respiratory infections. The infant was breastfed for the first month of life.

Shortly after vaccination, at 2,5 months of age, erythroderma and a generalized maculopapular rash appeared (**Figure 1**), followed by pneumonia, acute bronchiolitis, and a bacterial skin infection. His clinical condition progressively worsened, prompting referral for immunological evaluation. In the hospital, immunological tests revealed severe hypogammaglobulinemia, with undetectable levels of IgA and extremely low levels of IgG (0.32 g/l) and IgM (0.01 g/l). The patient exhibited marked eosinophilia, 35% (2.2 x10^3^/cells/uL), alongside a significant reduction in lymphocyte subsets, particularly T and B cells. T-cell analysis showed a low overall CD3+ T-cell count of 53% (764 cells/uL), with a near-complete absence of CD8+ cytotoxic T cells at 0,79% (11 cells/uL), leading to a high CD4/CD8 ratio (67.2). B cells were also severely reduced, while NK cells were relatively preserved at 42% (598 cells/uL). Immunophenotyping revealed a severely decreased CD19+ B-cell count (1.83%; <26 cells/uL), and 1,700–3,900 cells/uL), consistent with profound B-cell lymphopenia. The results are presented in **Tables 1**, **2**.

**Figure 1:**
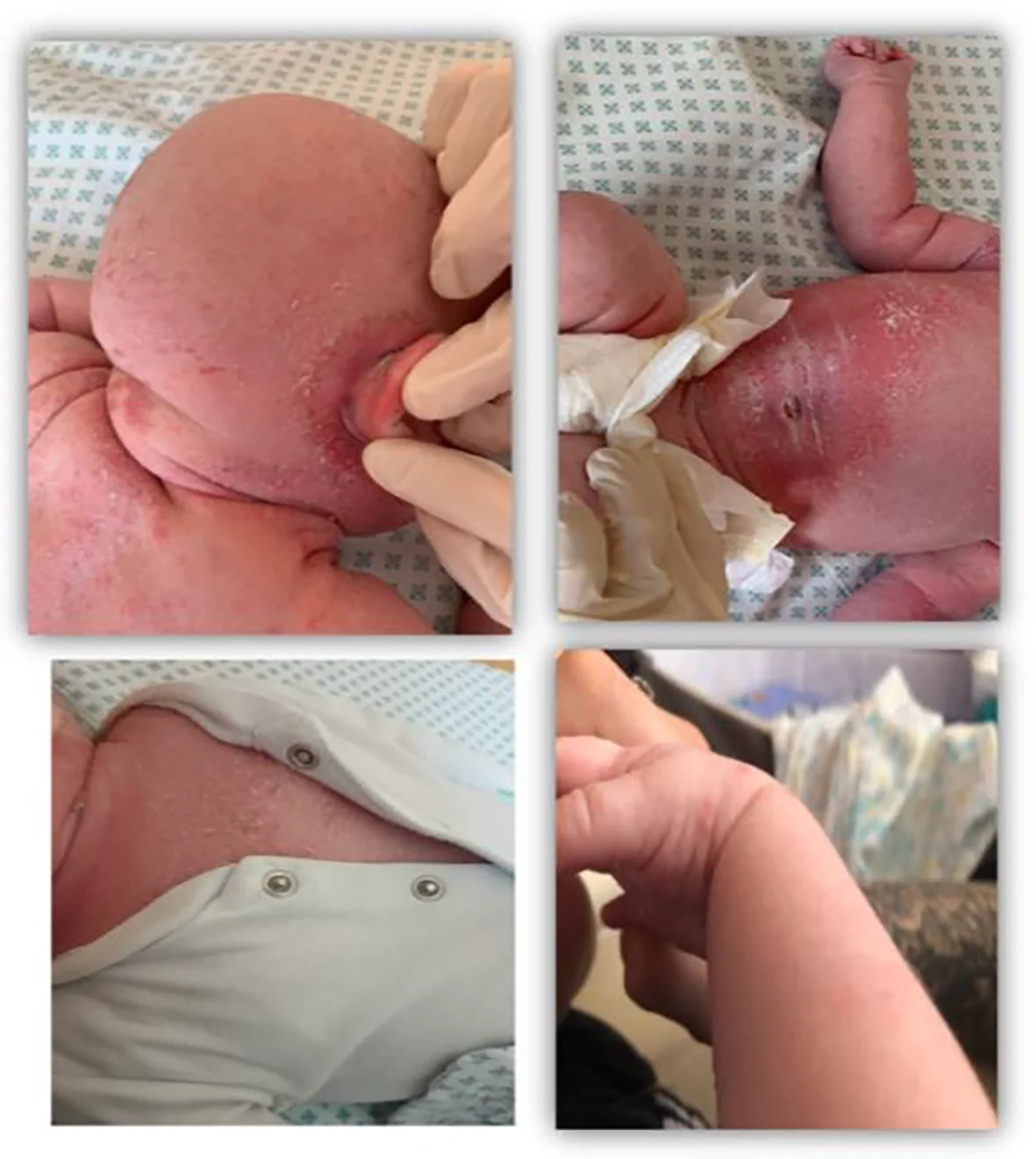
Maculopapular rash of the patient.

**Table 1:** Complete blood count.

Blood parameter	Result at 3 months	Reference range at 3 months	Result at 11 months after HSCT	Result at last follow-up (2 years 7 months)	Reference range at last follow-up (2 years 7 months)
White blood cells, count, x103/uL	6.16	6.51 – 13.32	3.08	6.10	5.14– 13.38
Reb blood cell count, x106/uL	4.26	3.43 – 4.8	2.73	5.20	3.89 – 4.97
Hemoglobin, g/dL	11.7	9.6 – 12.4	8.2	13.3	10.2 – 12.7
Lymphocytes, %	23.4	40.7 – 83.7	12.0	52.5	18.4 – 66.6
Lymphocytes, x103/uL	1.44	2.45 – 8.89	0.37	3.20	1.13 – 5.52
Monocytes, x103/uL	14.9	3.8 – 13.4	0.41	0.70	0.19 – 0.94
Platelets, x103/uL	779	224 – 529	27	138	202 – 403
Neutrophils, %	25.7	10.9 – 47.8	48.4	30.7	22.4 – 69
Neutrophils,x103/uL	1.58	0.97 – 5.45	1.49	1.88	1.54 – 66.6
Eosinophils, %	35.7	0 – 4	26.0	4.8	0 – 4.1
Eosinophils, x103/uL	2.20	0.03 – 0.61	0.80	0.29	0.03 – 0.53

**Table 2:** Immunological profile of the patient.

Immunoglobulins					
Name of test	Result, 3mo	Range	After HSCT, 11 mo	Result, 2y 7mo	Range
Serum IgA, g/L	<0.00	0 – 0.83	NA	0.14	0.2 - 1
Serum IgG, g/L	< 0.32	2.32 – 14.11	NA	7.42	4.42 - 8.95
Serum IgM, g/L	< 0.01	0 – 1.45	NA	0.49	0.19 – 1.46
Flow cytometry					
Lymphocyte populations	Result, 3 mo	Range	After HSCT, 11 mo	Result, 2y 7mo	Range
CD3+ T-cells, %	53.96	48 - 75	19.5	92.03	43 - 76
CD3+ T-cells,/uL	764	2300 – 6500	27	2944	900 - 4500
CD3+CD8+ T-cells, %	0.79	11 – 25	4	20.09	14 - 33
CD3+CD8+ T-cells,/uL	11	500 – 1600	5	642	300 - 1600
CD3+CD4+ T-cells, %	52.87	33 – 58	14	65.01	23 - 48
CD3+CD4+ T-cells,/uL	748	1500 – 5000	19	2080	500 - 2400
CD16/CD56+ NK-cells, %	42.21	2 – 14	49	3.16	4 - 23
CD16/CD56+ NK-cells,/uL	598	100 – 1300	67	101	100 - 1000
CD19+ B-cells, %	1.83	14 – 39	0	3.37	14 - 44
CD19+ B-cells,/uL	26	600 – 3000	0	108	200 - 2100
CD4/CD8 ratio	67.27	1.7 – 3.9	3.5	3.24	0.9 – 2.9

The immunological profile was atypical for SCID because CD3+ and CD4+ cells were present; however, the absence of CD8+ cells suggested a potential primary immunodeficiency.

Exome sequencing was performed using peripheral blood, and analysis of the inborn errors of immunity gene panel (including SNV/indel detection using DeepVariant and CNV detection using ExomeDepth) revealed a deletion, chr10[GRCh38]:g.14945090_15021878del, encompassing the *MEIG1* gene (OMIM:614174) and exons 1–3 of the *DCLRE1C* gene (OMIM:605988). The deletion had an observed-to-expected read ratio of 0.3, a borderline value between homozygous and heterozygous deletions. Exome sequencing quality control indicated 15% cell contamination with a female sample, which was also identified in freshly extracted blood samples on chromosomal microarray and confirmed to be of maternal origin by QF-PCR. Following exclusion of the maternal cell signal, the deletion was confirmed to be homozygous in the child, reinforcing the SCID diagnosis. Region of homozygosity (ROH) analysis, performed as part of our routine diagnostic workflow, identified no multiple ROH pattern, excluding parental consanguinity as the explanation for the homozygous state. The homozygous deletion of *DCLRE1C* exons 1–3 is consistent with the known mutational landscape of this gene: exon 1–3 deletions represent one of the most frequently reported CNVs in *DCLRE1C*, arising through homologous recombination with an upstream pseudogene (14, 15), and the carrier frequency of this deletion in our in-house cohort of individuals referred for indications other than SCID is 0.6%, supporting the plausibility of homozygous occurrence in non-consanguineous families. Karyotype culture failed to grow, preventing karyotyping - a phenomenon frequently observed with null variants of *DCLRE1C.*

SCID was genetically confirmed at 4 months of age. Until hematopoietic stem cell transplantation HSCT, the patient received antimicrobial prophylaxis and IVIG replacement (400 mg/kg every 4 weeks). Trimethoprim–sulfamethoxazole and oral fluconazole were continued until day −2 before HSCT, with fluconazole replaced by liposomal amphotericin B (2.5 mg/kg twice weekly) on day −1. No additional immunosuppressive therapy was given before conditioning.

At 7.5 months, a ~10 mm cyanotic infiltrate developed in the upper arm at the BCG vaccination site, as shown in **Figure 2**, accompanied by enlarged left axillary lymph nodes. Gastric lavage was negative for Mycobacterium tuberculosis DNA, while stool testing was positive, suggesting disseminated infection. Treatment with isoniazid, ethambutol, levofloxacin, and rifampicin was initiated; isoniazid was subsequently discontinued because of hepatotoxicity with elevated ALT and AST, and the regimen was modified accordingly.

**Figure 2:**
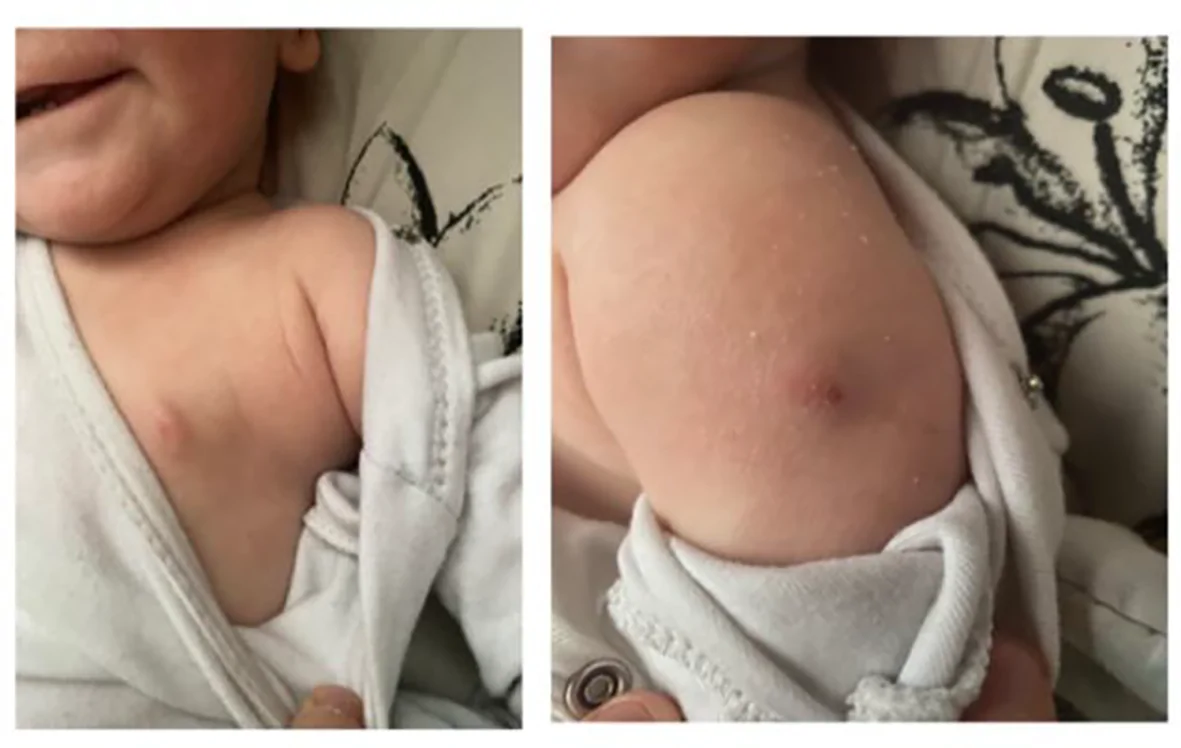
Cyanotic infiltrate of the patient.

In this historical case, antimycobacterial prophylaxis was not initiated immediately upon diagnosis because local experience with BCG-vaccinated SCID patients was very limited and no national prophylaxis protocol was available. This represents an important management limitation. Current evidence supports early antimycobacterial prophylaxis in BCG-vaccinated infants with SCID, even without clinical BCG disease, because of the high risk of local or disseminated BCG infection.

Once the diagnosis of SCID was confirmed, the patient was referred for allogeneic HSCT at Santaros Klinikos (Vilnius, Lithuania). At admission, the boy was 9 months old and continued antituberculosis treatment with Ethambutol, Levofloxacin, and Rifampicin. An allogeneic HSCT from a matched unrelated donor (10/10) was performed at 9.5 months of age. The patient received peripheral blood stem cells (8.0 x 10^6^ CD34^+^/kg, 1,9 x 10^8^ CD3^+^/kg) from a CMV-seronegative donor. A preparative regimen, consisting of Fludarabine 150 mg/m2, from day -7 to -3, and Treosulfan 30 g/m2, from day -6 to -4, was administered according to EBMT/ESID inborn errors working party guidelines (9). Graft-versus-host disease (GVHD) prophylaxis included Campath 1 mg/kg (from day -7 to -4), Cyclosporine A (CSA) targeted to 100–150 ng/mL, Mycophenolate mofetil (MMF) 15 mg/kg three times daily, from day +1 to day +34, and Methotrexate once daily on days +1, +3, and +6.

The patient was on continuous anti-tuberculous therapy with Levofloxacin, Ethambutol, and Rifampicin, which significantly interfered with CSA and prevented achievement of the therapeutic level (as shown in **Figure 3**).

**Figure 3:**
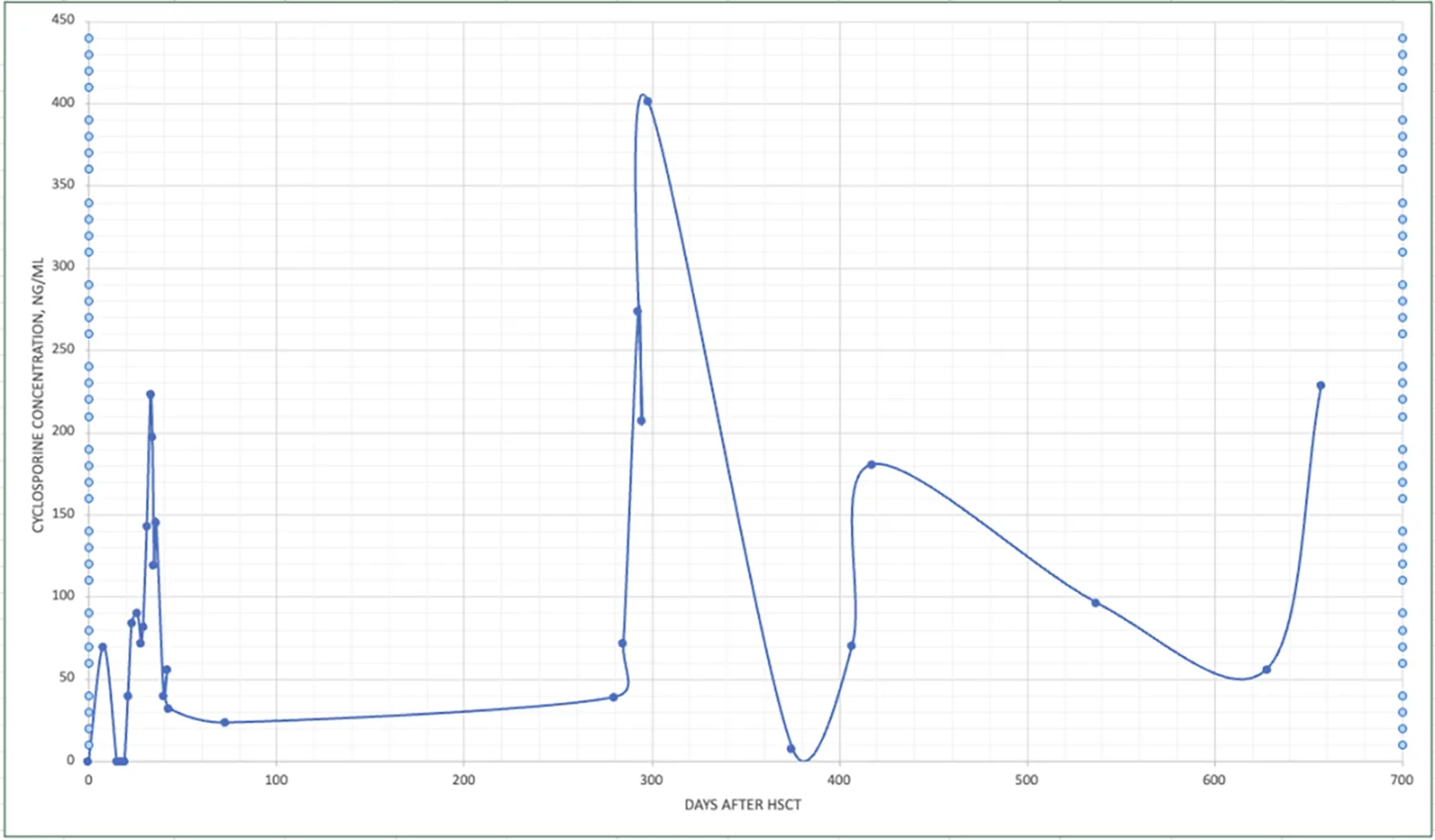
Cyclosporin levels after HSCT. The shaded rectangle marks the target cyclosporin level 100–150 ng/mL.

Neutrophil and platelet engraftment occurred on days +21 and +62, respectively. Before engraftment, on day +10 post-transplant, the patient developed fever and received empirical meropenem (160 mg IV three times daily for 9 days); blood cultures remained negative. After recurrence of fever following treatment discontinuation, meropenem was restarted for 8 days, and amikacin (100 mg IV twice daily) was added on day +27. On day +28, a painful axillary mass consistent with TBC lymphadenitis prompted addition of isoniazid (120 mg orally once daily), resulting in continuation of four anti-tuberculous drugs. Because of drug interactions, substantially higher CSA doses (up to 10 mg/kg orally twice daily) were required, although CSA levels remained below target (**Figure 3**). Grade I acute GVHD (skin++) developed on day +33 and responded well to topical steroids. From day +92 to +215, liver transaminases were markedly elevated (ALT 400–1100 U/L, normal <71 U/L; AST 400–1000 U/L, normal <96 U/L), possibly due to anti-tuberculosis therapy, and were managed with intravenous rehydration and ursodeoxycholic acid. The patient was discharged in stable condition on day +46 at 11 months of age, continuing ethambutol, levofloxacin, rifampicin, isoniazid, and CSA.

Chimerism analysis on days +28 and +62 showed 99% donor DNA in total leukocytes and CD3^+^, CD19^+^, and CD15^+^ populations. By day +204, mixed chimerism had developed, with donor DNA decreasing to 41.48% in total leukocytes, 62.15% in CD3^+^, 43.04% in CD15^+^, and 51.18% in CD19^+^ cells. By day +252, total donor chimerism had declined further to 18.56%, with the greatest recipient predominance in CD15^+^ and CD19^+^ cells (**Figure 4**). Because of progressive loss of donor chimerism, donor lymphocyte infusion was performed on day +296 using cryopreserved cells from the same donor (CD3^+^ 6.99 × 10^7^/kg; CD34^+^ 2.64 × 10^6^/kg). Thereafter, donor chimerism increased steadily, accompanied by quantitative immune-cell reconstitution.

**Figure 4:**
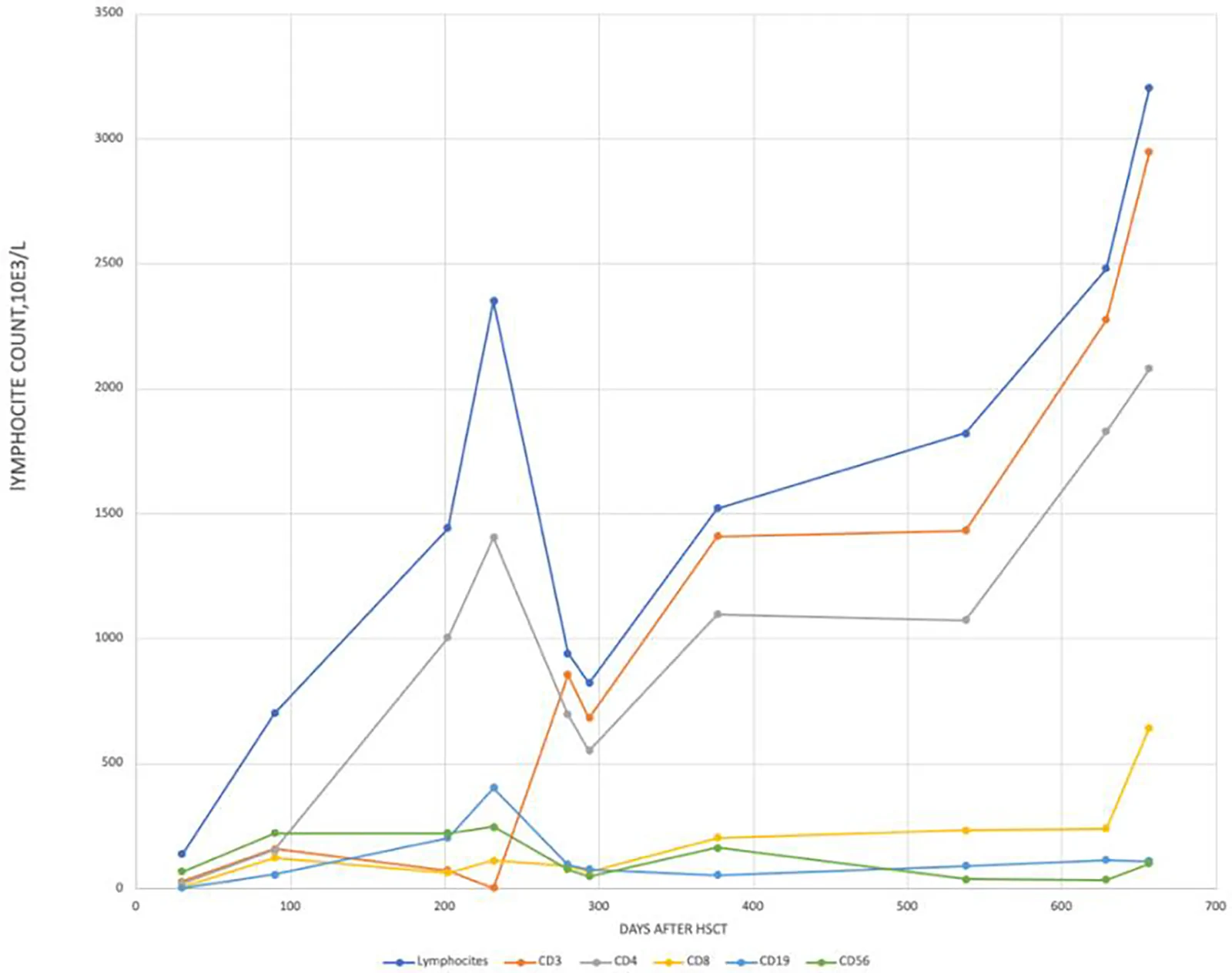
Lymphocyte count after HSCT. DLI – donor lymphocyte infusion performed on day +296.

Between days +184 and +215, the patient was hospitalized with severe tachypnea and hypoxemia (SpO_2_ 60%). Chest CT showed diffuse bilateral ground-glass and mosaic infiltrates with reduced aeration, consistent with interstitial lung disease. Bronchoscopy was normal; BAL (12 mL recovered after 30 mL saline instillation) was negative for bacterial, fungal, mycobacterial, and cytological abnormalities. EBV, CMV, and multiplex respiratory viral PCR were also negative. The patient improved with oxygen and antibiotics, although CT on day +273 showed persistent subpleural structural changes. Azithromycin three times weekly was started, with systemic prednisolone planned if deterioration occurred.

At 1 year 5 months, BCG-induced left axillary lymphadenitis worsened despite therapy, requiring drainage on day +228 and lymph node excision with local reconstruction on day +244. Smear confirmed *Mycobacterium tuberculosis* with no Rifampicin resistance. Delayed wound healing subsequently required reconstruction with latissimus dorsi and pectoralis muscle flaps on day +278.

Between days +245 and +276, serum Aspergillus galactomannan antigenemia was detected by ELISA-based assay. BAL was not performed because the parents declined inpatient evaluation and intravenous antifungal therapy. The patient received oral voriconazole for 5 days but was not receiving antifungal treatment at discharge. Between days +62 and +92, dilated cardiomyopathy with NYHA I heart failure was diagnosed and treated with furosemide, spironolactone, and carvedilol. Echocardiographic improvement between days +245 and +276 allowed discontinuation of furosemide and spironolactone, while carvedilol was continued. Red blood cell and platelet transfusions were required from days +31 to +154; thereafter, hemoglobin and platelet counts remained normal through day +184.

The patient was discharged on day +43 after HSCT, at 10 months and 29 days of age. Complete donor chimerism was confirmed on day +36 (99.19% donor overall, with complete donor chimerism in CD3^+^, CD15^+^, and CD19^+^ cells). After discharge, the patient remained clinically stable, with regular follow-up and adherence to treatment. At 1 year 10 months, antituberculosis therapy was discontinued after three consecutive urine and stool samples tested negative for *Mycobacterium tuberculosis* complex DNA. At 2 years 6 months, the patient remained clinically stable without recurrent mycobacterial infection or HSCT-related complications.

At 1 year 11 months, the latest chimerism analysis by real-time PCR showed mixed donor/recipient chimerism, with donor predominance in lymphoid compartments. Overall donor chimerism was 68.07% (recipient, 31.93%). Lineage-specific analysis revealed 95.54% donor chimerism in the CD3+ T-cell compartment (recipient, 4.46%), 85.00% in the CD19+ B-cell compartment (recipient, 15.00%), and 40.68% in the CD15+ myeloid compartment (recipient, 59.32%).

By 2 years 6 months, the underlying disease was clinically stable. Ongoing treatment included immunoglobulin replacement, antibacterial prophylaxis, iron and vitamin D3 supplementation, cardiomyopathy therapy, gastroprotection, symptomatic ibuprofen or acetaminophen as needed, and cyclosporine immunosuppression.

At the time of writing this report, the patient was 2 years and 8 months old. He is developing normally, meeting age-appropriate growth and developmental milestones, and undergoing regular blood tests and follow-up consultations with his treating physician.

IVIG replacement therapy was continued concomitantly with administration of inactivated vaccines; only live-attenuated vaccines were withheld. Assessment of vaccine-induced antibody responses was considered unreliable during ongoing IVIG replacement therapy because passively transferred antibodies may interfere with serological interpretation. The patient also received respiratory syncytial virus (RSV) immunoprophylaxis in accordance with Latvian national recommendations for infants at increased risk of severe RSV infection, given the underlying SCID and post-HSCT immunocompromised status. Regular monitoring for cytomegalovirus (CMV) reactivation was performed after transplantation, and all CMV PCR results have remained negative to date. The maternal T-cell population identified before HSCT was no longer detectable after transplantation, and donor-derived T-cell reconstitution was demonstrated during follow-up.

Despite mixed overall chimerism, the patient has remained clinically stable, without episodes of severe or recurrent infections after HSCT. At the most recent follow-up, he showed normal growth and age-appropriate psychomotor development. Continued clinical and laboratory surveillance is ongoing.

## 3 Discussion

We present the first documented case of severe combined immunodeficiency (SCID) in Latvia, caused by a homozygous null variant in the DCLRE1C gene encoding the Artemis protein. The main findings are threefold: genetic confirmation of Artemis-deficient SCID in a non-consanguineous Latvian family; an atypical T-cell profile obscured by transplacental maternal T-cell engraftment (TME); and a post-transplant course dominated by disseminated BCG disease and a clinically significant rifampicin–cyclosporine interaction. Together, these findings show how genetic, immunological, and pharmacological complexity compounded the risk to this infant, and support the value of the TREC-based newborn screening now implemented in Latvia.

Artemis deficiency, first described by Moshous et al. in 2001 (16), is caused by pathogenic variants in DCLRE1C, one of the seven most common genetic causes of typical SCID (1). The Artemis protein is essential for V(D)J recombination and DNA double-strand break repair, and its absence produces the characteristic T^−^B^−^NK^+^ immunophenotype and radiosensitivity (17).

Our patient’s presentation was further complicated by TME, reported in ~50% of SCID patients overall (18, 19) and in 42% of Artemis-deficient infants, compared with 100% in IL7Rα deficiency and 23% in RAG1/2 deficiency (20). Maternal cells can cross the placenta, and SCID infants lack sufficient immunity to reject them (20).

TME can be difficult to distinguish from Omenn syndrome because both may present with circulating T cells, erythroderma, lymphadenopathy, hepatosplenomegaly, and, as in our patient, eosinophilia (21, 22). Omenn syndrome results from hypomorphic RAG1, RAG2, or or Artemis mutations that permit some T-cell development but generate autoreactive, oligoclonally expanded T cells (21). Thus, diagnosis depends on confirming maternal rather than autologous T-cell origin. In our patient, this was established by peripheral-blood chimerism studies (chromosomal microarray and QF-PCR). Skin biopsy, T-cell functional assays, recent-thymic-emigrant enumeration, and CD45RO memory phenotyping were unavailable locally and could have strengthened TME characterization.

TME is clinically relevant because it increases the risk of post-HSCT acute GVHD (57.1% vs. 17.9%; relative risk 3.2) (20). Our patient developed grade I skin acute GVHD on day +33, consistent with this association. Because maternal T cells are post-thymic and TREC-negative, TME does not cause false-negative TREC screening; therefore, newborn screening would likely have detected SCID in this case (20).

The BCG-related complications observed fit a well-documented pattern for SCID: in the largest published cohort (349 BCG-vaccinated SCID patients, 28 centers, 17 countries), complications occurred in 51% (34% disseminated, 17% localized), with early vaccination (≤1 month) and a T-cell count ≤250/μL at diagnosis independently predicting complications (OR 2.1, 95% CI 1.4–3.4) (23). This last risk factor illustrates a specific hazard of TME in our patient: his CD3+ (764/μL) and CD4+ (748/μL) counts were within or near the reference range at diagnosis because of the circulating maternal T-cell population, even though his own T- and B-cell compartments were essentially absent — a count-based risk assessment at BCG administration would therefore not necessarily have flagged him as high-risk. Comparable burden has been reported in a Russian cohort (BCG infection in 58% of SCID patients specifically) (24), and in Iranian Artemis-deficiency data, where two of nine patients died of disseminated BCG disease despite treatment (25). In practice, though, the diagnosis could not reasonably have been suspected before vaccination: the infant was clinically healthy from birth to two months of age, and Latvia does not perform a complete blood count before BCG in the absence of clinical indications — underscoring the value of newborn screening in identifying SCID before symptom onset and live-vaccine exposure.

This case also highlights the consequences of delayed antimycobacterial treatment after SCID diagnosis. SCID was genetically confirmed at 4 months of age, two months after BCG vaccination, but prophylaxis was not initiated because no local protocol existed at the time. Disseminated BCG disease was diagnosed at 7.5 months, when multidrug therapy began, 5.5 months after vaccination. The subsequent course required prolonged antimycobacterial treatment, surgery for BCG lymphadenitis, and management of significant drug interactions.

Rifampicin markedly reduced cyclosporine A exposure through CYP3A4 and P-glycoprotein induction (26). Despite CSA escalation to 10 mg/kg twice daily, levels remained subtherapeutic, consistent with previous paediatric transplant reports (27). This relative under-immunosuppression may have contributed to declining donor chimerism observed between days +204 and +252 and the later need for donor lymphocyte infusion, supporting close therapeutic drug monitoring and, when feasible, rifamycin-sparing or rifabutin-based regimens during calcineurin inhibitor therapy (28).

National strategies to balance BCG-based tuberculosis prevention with SCID risk vary by tuberculosis burden and screening capacity. Saudi Arabia delayed BCG from birth to 6 months, reducing SCID-related BCG complications from 46.1% to 2.6% (29), while Taiwan similarly postponed vaccination (30). Other countries, such as Singapore, retain birth-dose BCG alongside TREC screening and prompt antimycobacterial prophylaxis for abnormal results (31). Latvia has adopted this latter model — TREC-based screening alongside continued birth-dose BCG since April 2023 - and had it been available at this patient’s birth, earlier diagnosis would likely have enabled prompt antimycobacterial prophylaxis, potentially preventing the disseminated disease that complicated his course.

Our patient underwent HSCT at 9.5 months while receiving treatment for active, incompletely controlled tuberculosis - a recognized predictor of worse survival, particularly beyond 3.5 months of age, with early deaths driven largely by infectious or pulmonary complications (10). Nevertheless, engraftment and long-term stabilization were achieved, with antimycobacterial therapy continued throughout HSCT in line with current guidance (23).

Overall, this case highlights the complex interplay among maternal T-cell engraftment, delayed diagnosis of Artemis-deficient SCID, BCG-related complications, and post-transplant management. In particular, the case emphasizes the importance of interpreting apparently preserved T-cell counts with caution when maternal engraftment is suspected and of anticipating clinically relevant drug interactions during concurrent antimycobacterial and immunosuppressive therapy.

## Conclusion

This first genetically confirmed case of SCID in Latvia illustrates how maternal T-cell engraftment can alter the clinical and immunological phenotype, delay diagnosis, and increase the risk of post-HSCT graft-versus-host disease, particularly in Artemis (*DCLRE1C*) deficiency. It also highlights the high risk of BCG-related complications in undiagnosed infants and the importance of early SCID detection. Nationwide TREC-based newborn screening, now implemented in Latvia, may help close this diagnostic gap by enabling earlier diagnosis, timely antimycobacterial prophylaxis, and optimal timing of transplantation while maintaining effective tuberculosis prevention strategies.

## Data Availability

The original contributions presented in the study are included in the article/**Supplementary Material**. Further inquiries can be directed to the corresponding authors.
