# Supplementary material for: Case report: Maternal engraftment of T-cells in a patient with Severe Combined Immunodeficiency due to DCLRE1C pathogenic variant - diagnostic and treatment challenges
Source: Front Immunol. 2026 Sep 30;17:1892927. doi: 10.3389/fimmu.2026.1892927 (PMC13661312; doi:10.3389/fimmu.2026.1892927)
Supplement: Supplementary file 1 [file DataSheet1.pdf]

Supplementary Figure 1 – Timeline

I NEWBORN

- Initially, a healthy boy born to a mother (G1 P1) with no history of a genetic disorder
- The patient was discharged from the hospital on the third day of life in good health condition

II 2 MONTHS OLD

- The patient was administered the BCG vaccine
- No fever or other reactions were observed after vaccination

III 2,5 MONTHS OLD

- The patient was admitted to a regional hospital due to maculopapular rash, wetting in axillary and groin regions, and erythroderma followed by Bacterial skin infection and Pneumonia

IV 3 MONTHS OLD

- The patient was transferred to the Children's Clinical University Hospital in Riga (CCUH), in moderate condition with Pneumonia and Bacterial skin infection (Staphylococcus aureus, Streptococcus viridans)

V IN THE CHILDREN'S CLINICAL UNIVERSITY HOSPITAL

- Symptoms of Acute bronchiolitis appeared: coughing, hoarseness, wheezing, and breathing with accessory muscles

VI IN THE CHILDREN'S CLINICAL UNIVERSITY HOSPITAL

- Based on the child's age, medical history, and recurrent bacterial respiratory and skin infections, a full examination was performed
- According to the examination and immunological tests, the recommendation is to monitor the patient and treat Prevention of infections: i/v Ig and Prophylactic a/b therapy: Sulfamethoxazole/ Trimethoprim

VII IN THE CHILDREN'S CLINICAL UNIVERSITY HOSPITAL
Genetic analysis was performed:

- Exome sequencing quality control of the Primary immunodeficiency gene panel
- CMA (Chromosomal microarray)
- KARYOTYPING AND FISH (Fluorescence in situ hybridization)

VII A: EXOME SEQUENCING QUALITY of the Primary immunodeficiency gene panel, Results:

- Revealed a deletion chr10[GRCh38]: g.14945090_15021878del, which includes *MEIG1* gene (OMIM:614174) and *DCLER1C* gene (OMIM:605988) 1-3 exons deletion
- The deletion has an observed/expected value of reads ratio of 0.2, which is a borderline value between homozygous and heterozygous deletion
- The exome sequencing quality control (QC) indicated 15% cell contamination with a female sample

VII B: CMA Results:

- Contamination was identified in freshly extracted blood samples using Chromosomal Microarray (CMA).
- QF-PCR confirmed that the contamination originated from the maternal source in both blood and skin biopsy samples.
- The deletion was confirmed to be homozygous in the child, reinforcing the **SCID diagnosis**

VII C: KARYOTYPING AND FISH Results:

- Karyotype culture failed to grow, preventin karyotyping—a phenomenon often seen in "null" variants of the *DCLRE1C* gene

VIII 4 MONTHS OLD

- Ongoing recurrent bacterial respiratory and skin infections, along with fungal infections
- Suggested treatment: **HEMATOPOIETIC STEM CELL TRANSPLANTATION (HSCT) as soon as possible**

XI 7,5 MONTHS OLD

- A cyanotic infiltrate in the upper arm, measuring approximately 10mm in diameter at the site of BCG vaccine administration, along with enlarged left axillary lymph nodes that have blurred borders
- MTB DNA analysis of the stool was positive, indicating a **generalized tuberculosis infection**

XII 9,5 MONTHS OLD - HSCT

- Hemopoietic stem cell transplantation (HSCT) was performed at **the Bone Marrow Transplantation Unit, Children's Hospital, Pediatric Oncology Center, Vilnius, Lithuania**
- The patient experienced a long and challenging post-transplantation period marked by an unstable immune condition

XIII 1 YEAR AND 1 MONTH OLD

- Patient presented with elevated hepatic transaminases, hepatosplenomegaly, oral candidiasis, and a maculopapular rash
- Progressive left axillary lymphadenopathy was diagnosed as BCG vaccine-related lymphadenitis, with further testing confirming **disseminated tuberculosis.**
- The patient developed secondary cardiomyopathy and is currently undergoing treatment for tuberculosis and heart failure

XIV 1 YEAR AND 5 MONTHS OLD

- The patient underwent an excision of the left axillary lymph node and several healing revisions, resulting in the wound being closed with a bleeding muscle flap

XV 1 YEAR AND 10 MONTHS OLD

- After three negative urine and stool tests for MTB DNA, the patient was discharged from tuberculosis treatment

XIII 2 YEARS AND 6 MONTHS OLD

- The patient's condition is stable concerning the underlying disease
- The patient has received immunosuppressive therapy, anti-inflammatory and immunomodulatory treatments, prophylactic antibiotics, iron replacement, vitamin D supplementation, treatment for cardiomyopathy, and gastroprotective therapy.
- CCUH specialists oversee observation and therapy, with a hemato-oncologist and a transplantation specialist at Vilnius Children's Hospital conducting online consultations
